# The Children's Ward

**Published:** 1887-05-14

**Authors:** 


### THE CHILDREN'S WARD.

The sun's rays blazed on the busy street,
There was rumble of carts and tramp of feet,
And men were shouting their penny wares,
While women talked over their household cares,
And everywhere seemed trouble and toil,
And endless hurry and great turmoil,
Save where a building silent and grand,
A little way back from the street did stand,
Studded with windows lengthy and bare,
And wearing a saddened but stately air.
Leaving the street we passed the gate,
Where a small crowd constantly seems to wait,
And along dark lobbies the East Wing toward,
We made our way to the Children's Ward.

There was sound of laughter and merry talk,
And the patter of little feet as they walk;
And all was cool, and airy, and light,
For the ward was long, and quiet, and bright.
Rows of small cribs as neat as could be,
Stretched just as far as the eye could see,
And little folks, clad in jackets red,
Were lying or sitting in every bed.
Nurses in caps and aprons of white,
Passed to and fro in the pleasant light,
Laying cool lotions on fevered head,
Or smoothing the ruffled untidy bed;
Feeding the hungry and quieting those
Who would wake the sleepy from needful doze,
Children with broken leg or arm,
Who have come to serious and sudden harm,—
Little ones injured by mother's sin,
Who in pain and grief must their life begin;
Older ones wounded by father's hand,
When he came home with a drunken band,—
Such have here found a haven of rest,
Where is neither rude rage nor obscene jest,
Where all is cleanly and pure and sweet,
And patience receives the reward that's meet;
Where boxes play music, and splendid toys
Are lent to the good little girls and boys;
Where pictures are hung above every bed,
And all with plentiful food are fed,—
From hunger, and want, and dirty homes,
Where few cared for their needs, or heeded their moans,
They have found their way to this pleasant place,
Where sympathy shines in each nurse's face.
Little ones here, when in extra pain,
Can comfort and peace and skilled help gain,
And the least of these is dear to God,
Who once as a child this sad earth trod.

That which is given to the Children's Ward,
Is given dear brethren, as unto the Lord.

